# #Healthy Selfies: Exploration of Health Topics on Instagram

**DOI:** 10.2196/10150

**Published:** 2018-06-29

**Authors:** Sachin Muralidhara, Michael J Paul

**Affiliations:** ^1^ Department of Computer Science University of Colorado Boulder Boulder, CO United States; ^2^ Department of Information Science University of Colorado Boulder Boulder, CO United States

**Keywords:** social media, Instagram, image sharing, topic modeling, computer vision, public health

## Abstract

**Background:**

Social media provides a complementary source of information for public health surveillance. The dominate data source for this type of monitoring is the microblogging platform Twitter, which is convenient due to the free availability of public data. Less is known about the utility of other social media platforms, despite their popularity.

**Objective:**

This work aims to characterize the health topics that are prominently discussed in the image-sharing platform Instagram, as a step toward understanding how this data might be used for public health research.

**Methods:**

The study uses a topic modeling approach to discover topics in a dataset of 96,426 Instagram posts containing hashtags related to health. We use a polylingual topic model, initially developed for datasets in different natural languages, to model different modalities of data: hashtags, caption words, and image tags automatically extracted using a computer vision tool.

**Results:**

We identified 47 health-related topics in the data (kappa=.77), covering ten broad categories: acute illness, alternative medicine, chronic illness and pain, diet, exercise, health care & medicine, mental health, musculoskeletal health and dermatology, sleep, and substance use. The most prevalent topics were related to diet (8,293/96,426; 8.6% of posts) and exercise (7,328/96,426; 7.6% of posts).

**Conclusions:**

A large and diverse set of health topics are discussed in Instagram. The extracted image tags were generally too coarse and noisy to be used for identifying posts but were in some cases accurate for identifying images relevant to studying diet and substance use. Instagram shows potential as a source of public health information, though limitations in data collection and metadata availability may limit its use in comparison to platforms like Twitter.

## Introduction

### Background

Social media can provide a vast source of insight into a wide variety of applications in public health monitoring and surveillance [1]. The bulk of social media-based health monitoring has relied on Twitter, a microblogging platform with over 300 million active users worldwide [2]. A wide variety of health topics are openly discussed on Twitter [3], providing researchers with a rich source of data for monitoring the spread of disease [4,5], dietary patterns [6,7], drug abuse [8,9], foodborne illness [10,11], and depression [12,13], among many other applications.

While Twitter has strengths as a data source, its dominance in research relative to other public platforms has been explained as a matter of convenience: Twitter provides free APIs to obtain large volumes of random or targeted samples of data [1]. However, microblogs are only one type of social media. Other social media platforms contain different types of data and are used in different ways, like sharing visual media. Image-sharing platforms, such as Flickr, Tumblr, Pinterest, and Instagram, are very popular; for example, Instagram, the most popular image-sharing platform, is more than twice the size of Twitter, with over 700 million active users [14]. Despite their popularity, relatively few public health studies have used these types of platforms as a data source [1].

Most prior health research using image-sharing platforms has focused on lifestyle issues, such as diet and substance use. Mejova et al [15] analyzed posts of food on Instagram, focusing on the relationship between food consumption and obesity. De Choudhury et al [16] also examined food consumption on Instagram, focusing specifically on dietary patterns in locations classified as “food deserts.” Yom-Tov et al [17] and Pless et al [18] examined imagery associated with eating disorders on Flickr and Tumblr, respectively. A few studies have looked at substance use on Instagram, including electronic cigarettes [19], marijuana [20], and opioids [21], as well as the marketing of substances on Instagram [22,23]. Garimella et al [24] looked more broadly at lifestyle choices in Instagram, including diet, physical activity, and drinking.

Many of these studies focused on the text features (eg, hashtags and captions) of the image posts, and some conducted a manual content analysis of the images themselves. Two of the studies cited above-used computer vision—a type of artificial intelligence that can automatically analyze the content of images—to perform automatic identification of certain types of images. Pless et al [18] built image classification models to identify images promoting anorexia, since such content may not be tagged with informative text captions. Garimella et al [24] attempted to estimate county-level health statistics from social media content and experimented with both text features and automatically-extracted image tags, finding that both types of information could be correlated with external health metrics.

This study seeks to characterize the health content shared on Instagram, the most popular image-sharing site, toward the goal of identifying potential areas of research that may benefit from this type of data source. In particular, we consider the following research questions: (1) what health topics are prominently shared in Instagram, and (2) what are the characteristics of those topics, specifically the types of images associated with the topics? This study is related to exploratory topic analyses of other platforms for health research [3,25], with an additional contribution of characterizing the features of images in addition to text. The dataset is made available as a resource to the public health informatics community.

## Methods

### Ethics Statement

This study was reviewed by the University of Colorado Boulder Institutional Review Board, which determined that it does not constitute human subjects research. However, given that publicly posted images may still be considered sensitive material by the users, we took steps to preserve privacy. We did not download any images as part of our study. Instead, we collected the URL pointers to images hosted on Instagram, which were processed by an external computer vision application programming interface (API). Our data collection only contains the abstract features extracted from the images.

### Data Collection and Preparation

While Instagram provides APIs for specific applications, Instagram does not provide an API to collect public data [26], in contrast to Twitter which provides widely-used streaming APIs [27]. We instead built a “crawler” that queries Instagram’s search engine, which returns the nine most recent posts matching a specified hashtag. The crawler accesses the webpage of the hashtag search engine, analogous to how a person would access the search engine in their browser. The page is downloaded, and the HTML is parsed to extract information such as the set of tags and the caption of the image.

We iteratively queried the search engine for 269 general health-related keywords that were used in previous work to obtain a general collection of health-related tweets [3]. The keywords were obtained from dictionaries of terms related to diseases, symptoms, and treatments, in addition to general words like “sick” and “health” that were added manually. The original keyword set contained over 20,000 terms, which were reduced to 269 words that were most common in Twitter, to conform to API limits on how many keywords can be searched. While developed for Twitter, we use the same list for Instagram here, as the list contains a broad set of terms that have previously been shown to be useful for collecting health-related social media posts.

Instagram only allows searching for hashtags rather than free text, so we treated each keyword as a hashtag (eg, “#flu” instead of “flu”). We repeated these 269 queries continuously from September 29, 2016, through October 25, 2016, attempting to simulate a “streaming” collection as with Twitter, and obtained 174,517 posts. We did not download the same post more than once as measured by a unique post identifier. However, if the same content was shared in multiple posts (eg, if multiple users shared the same image), these posts would be considered separate in the dataset.

Each post includes an image, a set of hashtags, and an optional free text caption. We used langid.py to identify and remove posts containing non-English captions [28]. We also removed hyperlinks, and nonalphanumeric characters. Stop words were removed using the natural language toolkit (NLTK) [29]. After filtering and processing, our dataset contained 96,426 documents posted by 77,327 users with an average of 1.25 posts per user.

We extracted “tag” features from each image using Microsoft’s Computer Vision API [30]. This service returns key phrase descriptors of images, such as “person” or “running”. The API was able to extract at least 1 tag for 79.24% (76,407/96,426) of images in our dataset. We refer to these tags as “image tags” to distinguish them from hashtags.

Once extracted, we treated the image tags as an additional type of text, along with captions and hashtags. In the final collection, there are 96,426 posts with a nonempty list of hashtags for an average of 15.2 tags per post. There were 95,208 posts (95,208/96,426; 98.74%) with nonempty captions for an average of 21.6-word tokens per caption. There were also 76,407 posts (76,407/96,426; 79.24%) with at least 1 image tag for an average of 3.7 tags per image. The dataset is shown in Multimedia Appendix 1.

### Topic Modeling

We use probabilistic topic models [31] to characterize the major themes of health-related discussion in Instagram. Topic models are tools for clustering related words into themes or concepts called “topics” and for identifying the topic composition of documents. Topic models have been used in health research as a method of performing content analyses of large datasets [25,32-34].

A topic model is a statistical model with many latent variables and parameters that can be inferred by fitting the model to data. In this model, each “topic” has a probability distribution over words, estimated from data, and topics are usually represented by presenting the 10-20 most probable words in the topic. Additionally, each document has a probability distribution over topics, which can be used to characterize the topic composition of a document and to identify documents that describe particular topics.

Topic models take documents, represented as vectors of word counts, as input. The model parameters (ie, the distribution over topics *θ*_d_ in each document *d*, and the distribution over words *ϕ*_k_ for each topic *k*) are estimated to fit the observed data (ie, the word counts in each document). The estimated parameters are often interpretable to people, and the words associated with each topic cluster can be used to assign a concept to the topic [35].

#### Polylingual Topic Model

The polylingual topic model [36] is an extension of a traditional topic model that is applied to multiple languages. This model can be used for datasets in which documents have multiple versions in multiple languages. For example, translations of a document into other languages, or articles in different languages that are known to be about the same topic, like different versions of Wikipedia articles. In the polylingual topic model, the distribution over topics *θ*_d_ is shared across all versions of the document, while each topic has a different distribution over words specific to each language *l*, *ϕ*_lk_*.*

In this work, we treat the different modalities of data—captions, hashtags, and image tag features—as different “languages” and apply the polylingual topic model to these 3 types of data. That is, each topic has a distribution over caption words, a distribution over hashtags, and a distribution over image tags. This will provide different views of each topic, allowing us to leverage multiple types of data and provide a complete understanding of the topics.

#### Model Estimation

We used the Polylingual Topic Model implementation from MALLET [37]. The hyperparameter for the topic distribution prior (ie, “alpha”) was set to 1.0, and we used the default algorithm settings. The number of topics was set to 150. The model does not require each document to have a version in all 3 “languages,” and if a document did not contain a caption or image tags, we still included the document but without those data types.

Because the topic model output in this study is interpreted qualitatively to be used in a content analysis, we also used qualitative judgment in performing model selection [1]. To avoid extensive model selection, we relied on default hyperparameters for the model. To choose the number of topics, we compared the output with 50, 100, and 150 topics. We selected 150 topics because this setting provided topics that were qualitatively more coherent.

#### Topic Identification

After running the topic model, we examined the 20 most probable words in each “language” of each topic. The two authors independently annotated each topic, labeling each topic with a phrase that describes the group of words or marking the topic with an “unknown” label if the words do not form a coherent theme. The annotators then discussed the independent labels with each other to determine if the 2 labels described the same concept (eg, the free text labels could be similar but different strings, such as “Running” and “Jogging”), and to decide on a final label.

When comparing whether the 2 annotators thought a topic was coherent, as opposed to the “unknown” label, the annotators agreed on 124/150 (82.7%) of the topics (Cohen kappa=.62). When comparing whether the 2 annotators thought a topic was related to health, the agreement was 136/150 (90.7%) with Cohen kappa=.77.

Additionally, we grouped the topics into coarse-grained categories, to make the results easier to summarize. One annotator created a grouping of the topics and then iterated with feedback from the other annotator. Categories are not mutually exclusive; topics could be assigned to more than 1 category.

### Topic Analysis

The topic model gives the probability of each topic *k* in each document *d*, *θ*_dk_*.* To get the overall prevalence of each topic, we calculate the average proportion, where *D* is the number of documents: 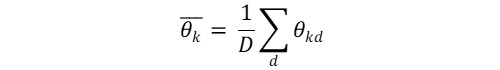
To estimate the prevalence of the coarser categories, we simply define each category’s prevalence of the sum of its topic proportions, where *C*_j_ is the set of topics in category *j*: 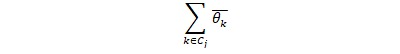
To summarize the degree to which 2 topics have a tendency to occur together in documents, we calculate the Pearson correlation of the *θ*_dk_ values for all pairs of topics. Most of the topic pairs with high correlations were similar topics in the same category. To discover less obvious topic co-occurrences, we focus only on topic pairs from different categories.

## Results

Of all the topics, 93/150 (62%) were identified as coherent, with 47/150 (31%) related to health and 46/150 (31%) not related to health. The 47 health topics were grouped into 10 high-level categories: acute illness, alternative medicine, chronic illness and pain, diet, exercise, health care & medicine, mental health, musculoskeletal health and dermatology, sleep, and substance use.

Examples of health topics are shown in Table 1, while the complete set of 47 health topics organized across the 10 categories is provided in Multimedia Appendix 2. Examples of nonhealth topics are shown in Table 2. As can happen with unsupervised topic models, many clusters have very similar and overlapping content, with similar or identical names given by the annotators.

Comparing the different modalities, hashtags tend to contain words specific to the topic (eg, “cancer,” “diabetes,” “allergies”), while caption words give indications of the context of the posts (eg, “feel,” “love,” “hope,” “proud”). In some cases, the caption words include first-person (eg, “i’ve,” “i’ll”) and informal (eg, “awesome,” “lol”) language that might be observed in personal conversations. Other topics include caption words consistent with advertising (eg, “product,” “call,” “email,” “consultation”).

Examining the image tags, there are often not many tags that are directly related to the health topics, with the exception of topics related to food and beverage, which usually had explicit image descriptors of the corresponding food. For example, the *Meat* topic contains the image tags “meat” and “barbeque,” *Desserts* contains “dessert” and “chocolate,” *Alcohol* contains “alcohol” and “beer,” and *Caffeine* contains “coffee” and “coffee cup.” A small number of other topics are also associated with image tags that are directly related to the topic: *Sleep* includes “pillow” and “bed,” and *Dental health* includes “toothbrush” and “mouth.”

**Table 1:** The top 10 words in each modality (hashtags, caption words, and image tags) for 6 example health topics.

Modality	Bodybuilding	Cancer	Caffeine	Desserts	Insomnia	Suicide & self-harm
Hashtags	bodybuilding shredded muscle gym abs physique veins gymlife bodybuilder gains	cancer cure chemo breastcancer cancersucks breastcancerawareness pink chemotherapy fuckcancer hope	caffeine coffee coffeelover coffeeaddict coffeetime coffeeholic hot drink cafe coffeegram	food cravings foodporn delicious chocolate foodie yummy dessert sweet yum	insomnia bedtime workout art selfie rest night natural sleepy amazing	anxiety depressed sad suicide suicidal depression cutting sadness broken selfharm
Captions	bro man nice work likes gym muscle hard follow training	cancer breast awareness month pink support women chemo fight family	coffee day tea today love drink cup hot green feeling	chocolate cream pumpkin eat good made butter ice cake peanut	sleep night bed hours time back asleep sleeping make nights	don’t feel talk people hate i’ve stop anymore fucking cry
Image tags	man underpants sport indoor barbell cellphone phone holding exercisedevice swimsuit	group standing people beautiful crowd little girl wearing white pink	beverage food coffee table drink coffeecup breakfast pastry dixiecup doughnut	dessert chocolate slice food piece eaten cream plate fork pastry	indoor lying bedclothes smiling pillow blanket sofa bedroom glasses cloth	close dark woman staring clouds hand cloudy road nightsky mountain

**Table 2:** Examples of topics that are not directly about health.

Modality	Inspiration	Poetry & quotes	Spirituality	Politics	Cats	Grunge/emo
Hashtags	inspiration selflove happiness recovery positivity positivevibes loveyourself heal hope positive	poetry quotes pain words quote writer love writersofinstagram hurt writing	heal healing energy meditation love spiritual soul mind spirit light	trump vaccines nature vegan wakeup hillary blacklivesmatter usa clinton organic	catsofinstagram cat cute cats kitty kitten pet meow fluffy animal	grunge tumblr emo alternative depressed depression goth sad aesthetic punk
Captions	life things live past true grateful time living mind people	love heart words world soul make life mind hurt give	energy healing body soul light life heart deep space love	people world trump media american america vote country drugs government	feel hope poor baby glad aww cat hear rest sick	don’t feel i’ll back i’ve make yeah hope feeling man
Image tags	indoor posing sky rock garden water mountain bushes clouds can	wearing day mammal dark shore grass open plaque building abstract	posing person fresh forest mountain mammal silhouette sunset sign distance	indoor screen display electronics flat suit sign text newspaper computer	domesticcat mammal laying animal sleeping cat white rodent grey gray	indoor close person young hair blue glasses looking messy silhouette

While not explicitly about the health topic, some topics contain image tags that convey other characteristics of the imagery. For example, the image tags of the *Mental health* topic suggest positive imagery (eg, “smiling” and “nature”) while the tags of the *Suicide & self-harm* topic suggest negative imagery (eg, “dark” and “cloudy”). The *Cancer* topic contains imagery associated specifically with breast cancer awareness, with the tag “pink” appearing as an image tag, as well as in the top hashtags and caption words.

A common theme across topics is that images frequently contain people. The image tag “posing” is the top tag associated with 11 topics (ie, *Chronic illness*, *Diabetes*, *Gym/fitness*, *Health care*, *Nursing*, *Hospitalization*, *Mental health*, *Skin health*, *Tanning*, *Cosmetic surgery*, *Dental health*). Other topics have the top tag of “person” (ie, *Illness*), “group” (ie, *Cancer*), “woman” (ie, *Headaches & body aches*), and “man” (ie, *Bodybuilding*). The image tags “swimsuit” and “underwear” are especially common in many of the exercise and fitness topics. This matches an observation in a previous study of fitness images on Instagram which found that “most images contained posed individuals with some degree of objectification” [38].

Gender associations can also be observed in the image features. There are four topics that contain a male-associated image tag (ie, “man,” “boy,” “male”) but no female tag (ie, *Diabetes*, *Massage*, *Gym/fitness training*, *Bodybuilding*), and 6 topics that contain a female-associated tag (ie, “woman,” “girl,” “female”) but no male tag (ie, *Cancer*, *Musculoskeletal pain*, *Headaches & body aches*, *Exercise*, *Gym/bodybuilding*, *Gym/fitness*). Only 1 topic (ie, *Allergies*) included both genders in the top 10 image tags (ie, “woman” and “boy”).

In one case, 2 topics with very similar text features had different gender patterns in the images. Consider the topic with top hashtags, “workout,” “fitness,” “gym,” “fit,” “exercise,” and top caption words, “week,” “day,” “workout,” “work,” “good;” and a similar topic with hashtags, “workout,” “abs,” “gym,” “muscle,” “chest,” and caption words, “work,” “back,” “legs,” “leg,” “strong.” These top words do not explicitly contain gendered words, but in the image tags, the former contains “woman” while the latter contains “man” and “male.” Table 3 shows the 10 topics with the highest average proportions in documents, and Table 4 shows the cumulative proportions of each of the 10 categories.

**Table 3:** The 10 most prevalent individual topics in the dataset, ranked by their average topic proportion out of 150 total topics. When multiple topics have the same name, we show the integer index of the topic in parentheses to distinguish them.

Topic	Average Probability
Suicide & self-harm	0.012
Bodybuilding (Topic 135)	0.010
Exercise	0.009
Healthy food (Topic 2)	0.009
Gym/bodybuilding (Topic 14)	0.009
Marijuana	0.008
Healthy food (Topic 67)	0.008
Vitamins & supplements	0.008
Skin health	0.008
Gym/fitness	0.008

**Table 4:** The topic categories ranked by prevalence, where each category’s prevalence is defined by the sum of the individual topic proportions of the category’s topics.

Topic Category	Cumulative Probability
Diet	.086
Exercise	.076
Musculoskeletal health & dermatology	.046
Alternative medicine	.042
Chronic illness & pain	.039
Health care & medicine	.033
Mental health	.026
Substance use	.021
Sleep	.013
Acute illness	.012

Topics about diet and exercise are by far the most prevalent topics, while topics about acute illness and sleep are uncommon. Topics with high probabilities tended to be more coherent than low-probability topics. Of the topics with the highest probabilities, 24/25 (96%) were labeled as coherent by the annotators. Of the topics with the lowest probabilities, only 15/25 (60%) were labeled as coherent. The variability in average probabilities was low; the values ranged from .005 to .012.

Table 5 shows the 10 pairs of health topics with the highest correlations. Some of the strongest correlations are with the *Vitamins & supplements* topic, which co-occurs with a variety of other health topics. Table 6 shows the most correlated 10 topic pairs such that one topic is a health topic and the other is a nonhealth topic. Inspirational and supportive topics (ie, *Inspiration* and *Poetry & quotes*) tend to co-occur with mental health and exercise topics, and topics about religion and spirituality tend to co-occur with certain health topics, like *Alternative medicine* and *Yoga*.

**Table 5:** The 10 most correlated pairs of health topics.

Topic pair		
Topic A	Topic B	Pearson Correlation
Vitamins & supplements	Energy & hydration	.138
Vitamins & supplements	Health science	.131
Vitamins & supplements	Headaches & body aches	.121
Energy & hydration	Headaches & body aches	.067
Vitamins & supplements	Skin health	.060
Chronic illness	Mental health	.057
Chronic illness	Hospitalization	.050
Alternative medicine	Health science	.050
Running & cardio	Injuries & rehabilitation	.049
Headaches & body aches	Massage	.047

**Table 6:** The 10 most correlated pairs of topics, where each pair contains 1 health topic and 1 nonhealth topic.

Topic pair		
Topic A	Topic B	Pearson Correlation
Suicide & self-harm	Grunge/emo	.107
Mental health	Inspiration	.081
Hospitalization	Cats	.080
Vaccination	Politics	.063
Hospitalization	Religion/Christianity	.059
Yoga	Spirituality	.056
Alternative medicine	Spirituality	.048
Fitness training	Sexuality	.048
Suicide & self-harm	Poetry & quotes	.043
Gym/fitness	Inspiration	.036

## Discussion

### Principal Findings

The topic model results show a large and diverse set of health topics are discussed in Instagram. Qualitatively, we find that the top hashtags tend to be the best descriptors of topics, while caption words give some indication of what kind of messages are associated with the topics, such as whether they are more informational or conversational. The extracted image tags are generally much less coherent, though they do help characterize the types of images that are associated with each topic. For example, many of the topics related to pain contain images of animals, perhaps because users post cheerful images in response to pain. The tag “posing” appears in some topics, suggesting these posts may be informational rather than personal. The *Cancer* topic contains the image tags “group” and “crowd;” it appears these many posts in this topic are about cancer awareness events. In some cases, image tags were the defining characteristics that distinguished clusters that were otherwise very similar, which suggests that images are informative beyond the hashtags and captions to conduct content analyses of Instagram posts.

Qualitatively, it appears that in most cases the image tags are not specific enough to be useful for directly identifying posts relevant to a specific health application. However, tags of food and beverages appear to be fairly specific and accurate, suggesting that computer vision may help in identifying posts for studies of diet and food consumption. The only previous work we are aware of that used automatically extracted image tags for this purpose is [24], which found that image tags were predictive of lifestyle factors; for example, “glass”, “liquid” and “beverage” were associated with alcohol consumption. The authors suggested that image tags may be useful for identifying stigmatizing behaviors, where social media users may post images of an activity but not explicitly tag the activity. Even nonstigmatized activities, like general food consumption, may not be tagged by a user in a way that is specific enough to identify by text search, while computer vision may help. We observed that image tags extracted from the computer vision API did not usually identify a specific dish, but could at least identify broad categories, like “meat” and “vegetable,” and in some cases were more specific, like “potato” and “doughnut.” We, therefore, argue that this type of computer vision tool can expand the amount of data available for studying patterns in food consumption.

We gained additional insights by considering the co-occurrences of health topics in the data. For example, the *Vitamins & supplements* topic is less likely to appear in a post in isolation but instead co-occurs with other topics, likely because supplements are discussed in the context in which they are used. Using this data to study nutrition in a population may, therefore, be able to show how nutrition is discussed and applied to specific aspects of health. Some pairs of topics with high correlations may indicate comorbidities, such as *Chronic illness* and *Mental health* [39].

Co-occurrences with nonhealth topics may give insights into other contexts in which health is discussed. We observed that many health topics frequently co-occur with inspirational topics, such as topics containing poetry and quotes, or topics about nature, as well as topics related to spirituality and religion. These types of posts may give insight into how individuals cope with and support others with, illness and disease.

An additional observation in some topics is the use of certain hashtags to identify a specific community of users [40], such as the #wlscommunity in the weight loss topics. Online health communities have been studied to understand social support and behavior change in managing health conditions [41]. Instagram-based communities may be a unique source for studying similar issues. Communities for specific demographic groups (eg, #girlswholift) are also present. In some cases, demographic associations could be gleaned from the image tags, even if the text tags were not explicitly gendered.

One of our methodological contributions was to repurpose an existing tool, the polylingual topic model, for a new task of combining different modalities of data in a topic model. We showed that automatically extracted image tags from a computer vision API can be treated as text tokens in an existing topic model. Beyond topic models, our observations of the results suggest that these extracted image tags are in some cases useful descriptors of images. We suggest that this type of tool can be applied to images for health research more broadly.

We observed the same broad set of topics in Instagram that have previously been seen in Twitter [3], suggesting that Instagram could serve as a potential data source for many of the same applications for which Twitter is used. Moreover, the presence of the first-person language (eg, “i’ve”) in some topics indicates that health posts on Instagram include personal health mentions, which is an essential characteristic for some types of surveillance [42]. This has implications for social media-based health surveillance because this suggests that Instagram could be used as a data source for similar areas of research, while having the potential benefit of covering a larger population than Twitter. We do not suggest that one platform is universally better than another, but instead, using data from multiple platforms can result in better surveillance than reliance on one platform [43,44].

Instagram may complement Twitter as a data source because it has a different demographic distribution. The user base of Instagram is younger, lower income, and more urban compared to Twitter, [45]. These demographics cover populations that are traditionally harder to reach in health research [46], and so Instagram may be well-suited for studying such populations. This argument has been made for using Twitter [47], yet Instagram has an even heavier bias toward these populations. Additionally, Instagram has a gender bias that Twitter does not have, being nearly 50% more popular among women than men [45].

### Limitations

Not all health topics are discussed widely on Instagram, which may be a limitation of using Instagram. By far the most common topics in Instagram are related to diet and exercise, while topics on acute illness, which would be needed for a task like influenza surveillance, are the least common. This may explain why all prior work we identified using image-sharing platforms for health research was related to lifestyle factors, such as diet (most common) and physical activity. Nonetheless, topics about infectious disease do exist on Instagram, and so it may be worth investigating the utility of contributing this data to an ensemble surveillance system [43]. To the best of our knowledge, no prior work has studied Instagram for infectious disease surveillance, which would be a good candidate for future research. However, this study did not collect Instagram posts from a large enough span of time to validate the data for such a task.

Another limitation of using Instagram is the limited availability of metadata. When crawling Instagram, it is difficult to sample data uniformly across time, as Instagram does not provide a streaming API analogous to Twitter’s widely-used APIs, which would make it difficult to extract the long-term pattern, for example, to validate influenza tracking. Location data also appears to be difficult to obtain when crawling from the web. In our dataset, 46% of posts contained a user-specified location string, but these were not in a standard format, and many of them were names of businesses or other specific locations, without reference to a geographic area. Geolocation from Instagram is less well understood in social media research, as well as inference of other demographic attributes that may be important in public health research. Richer data may be available from certain resellers; for example, Gnip, who is the official seller of Twitter data, also sells Instagram data, which can be searched by either tag or geolocation.

In addition to limitations of this data, there are limitations with the topic model methodology. Topic model evaluation is notoriously difficult [35], though research has found that this methodology can provide overlapping insights with more traditional, manual text analysis [48]. There is subjectiveness in choosing the number of topics and labeling and categorizing the topics, which we mitigated by having two researchers involved in each step. An advantage of the topic modeling approach for this study is that it can be applied to the entire dataset of nearly 100,000 posts, and the word distributions highlight the features associated with each topic across the three modalities. Furthermore, with such a large number of topics in the data (ie, 47 health topics identified by the approach used here), a typical sample size for manual content analysis, on the order of 1,000 posts [19-21], would be insufficient for accurately learning the prevalence of each topic.

Another limitation of topic modeling is that the topics characterize *what* is being discussed, but it is difficult to describe *how* the content is presented. For example, the topic model can identify posts that are related to marijuana, but it does not distinguish between personal marijuana use, information about marijuana, or advertisements for cannabis products—distinctions that have been made in prior work using more qualitative methods [20]. However, the topic model is still an essential first step of filtering and retrieval, after which topic-specific posts could be analyzed in more depth.

We note that there exist other methods for identifying thematic patterns in text beyond probabilistic topic models that have been used in health research, such as network-based clustering on term co-occurrence graphs [49]. Most such methods, including topic models, rely on co-occurrence statistics of words and have similar properties and limitations. We used the polylingual topic model due to its ability to integrate different “languages” or modalities of data.

Finally, the grouping of topics into ten overlapping categories is also limited. Some topics were difficult to categorize, and the boundaries of some categories were difficult to define. However, the goal of the categorization is to present the raw results (available from our dataset in Multimedia Appendix 1) more concisely. The mapping of topics to categories is transparent (viewable in Multimedia Appendix 2) so that the results can be interpreted correctly.

### Conclusion

This study shows that health is discussed on Instagram in a variety of ways, and there is potential for computer vision techniques to automatically characterize health-related images, which could extend public health surveillance of social media beyond text-based analysis. Our dataset of nearly 100,000 posts is available to allow for the study of specific topics and image tags in more depth. There are pragmatic reasons why this popular platform has been used in research relatively little compared to platforms like Twitter and Facebook, but our results and discussion point to ideas that image-sharing platforms like Instagram may complement other social media data sources in health research.

## Appendix

Multimedia Appendix 1 The dataset of 96,426 Instagram posts. The raw data is not included for privacy reasons, but can be collected through the URLs provided. The dataset includes the additional information inferred for each post: the image tags, and the topic model probabilities. Descriptions of all 150 topics are also included, as well as the 269 keywords used to search for posts.

Multimedia Appendix 2 The descriptions of the 47 health topics. Each slide contains a table corresponding to one of the ten health categories, along with the top ten hashtag, caption, and image features for each topic in that category. Some topics have the same name, in which case we added the topic index (1-150) in parentheses to the name to differentiate these topics.

## Abbreviations

API: Application Programming Interface
NLTK: Natural Language Toolkit
