# Supplementary material for: #Healthy Selfies: Exploration of Health Topics on Instagram
Source: JMIR Public Health Surveill. 2018 Jun 29;4(2):e10150. doi: 10.2196/10150 (PMC6045785; doi:10.2196/10150)
Supplement: Multimedia Appendix 2 [file publichealth_v4i2e10150_app2.pdf]

Acute illness

	Illness	*Allergies*
**Hashtags**	health<br>flu<br>sick<br>instalike<br>green<br>nofilter<br>white<br>day<br>relax<br>fresh	allergies<br>asthma<br>allergy<br>hayfever<br>itchy<br>allergic<br>sick<br>sneezing<br>spring<br>sneeze
**Caption**	week<br>work<br>days<br>sick<br>miss<br>season<br>weeks<br>flu<br>bring<br>weather	allergies<br>asthma<br>allergic<br>allergy<br>itchy<br>year<br>weather<br>inhaler<br>eyes<br>reaction
**Image**	person<br>hair<br>young<br>nature<br>colored<br>baby<br>red<br>dress<br>jacket<br>maple	indoor<br>people<br>woman<br>soup<br>brown<br>field<br>dog<br>variety<br>boy<br>grassy

Alternative medicine						
	Alternative medicine	*Natural remedies (40)*	*Natural remedies (104)*	*Massage*	*Vitamins & supplements*	*Yoga*
**Hashtags**	acupuncture<br>health<br>wellness<br>healing<br>holistic<br>yoga<br>chinesemedicine<br>medicine<br>tcm<br>natural	essentialoils<br>natural<br>organic<br>wellness<br>holistic<br>doterra<br>aromatherapy<br>allnatural<br>momlife<br>chemicalfree	sick<br>healthy<br>natural<br>remedy<br>ginger<br>fever<br>nausea<br>clean<br>fall<br>florida	massage<br>pain<br>relax<br>arthritis<br>aches<br>therapy<br>wellness<br>painrelief<br>tension<br>relaxation	vitamins<br>health<br>wellness<br>energy<br>vegan<br>detox<br>nutrition<br>supplements<br>guthealth<br>natural	yoga<br>breathing<br>yogi<br>balance<br>yogaeverydamnday<br>meditation<br>namaste<br>ear<br>breathe<br>stretch
**Caption**	health<br>medicine<br>healing<br>acupuncture<br>natural<br>herbal<br>symptoms<br>herbs<br>chinese<br>heal	oil<br>oils<br>essential<br>blend<br>drops<br>cold<br>lavender<br>peppermint<br>support<br>respiratory	sick<br>feel<br>ginger<br>morning<br>time<br>throat<br>pain<br>sore<br>food<br>made	pain<br>massage<br>body<br>muscle<br>muscles<br>relief<br>deep<br>arthritis<br>sore<br>aches	health<br>system<br>body<br>immune<br>vitamin<br>healthy<br>benefits<br>helps<br>natural<br>vitamins	yoga<br>body<br>love<br>breathing<br>mind<br>class<br>breath<br>practice<br>pose<br>breathe
**Image**	indoor<br>red<br>animal<br>food<br>forest<br>drink<br>arranged<br>wood<br>wooden<br>ceiling	several<br>food<br>hand<br>mammal<br>soup<br>drink<br>baby<br>wood<br>holding<br>beer	laying<br>white<br>bed<br>cup<br>yellow<br>coffee<br>dish<br>beautiful<br>half<br>garden	indoor<br>close<br>man<br>floor<br>decorated<br>wearing<br>half<br>hotel<br>honey<br>cake	beverage<br>vegetable<br>indoor<br>fresh<br>plant<br>arranged<br>plastic<br>fruit<br>different<br>fruitdrink	beautiful<br>indoor<br>shore<br>tree<br>water<br>beach<br>floor<br>park<br>feet<br>silhouette

Chronic illness & pain

	Chronic illness	*Cancer*	*Diabetes*	*Musculoskeletal pain*	*Headaches*	*Headaches and body aches*
**Hashtags**	chronicillness<br>spoonie<br>chronicpain<br>invisibleillness<br>fibromyalgia<br>arthritis<br>autoimmunedisease<br>butyoudontlooksick<br>chronicfatigue<br>lupus	cancer<br>cure<br>chemo<br>breastcancer<br>cancersucks<br>breastcancerawareness<br>pink<br>chemotherapy<br>fuckcancer<br>hope	diabetes<br>type<br>diabetic<br>inspiration<br>wellness<br>diabetesawareness<br>insulin<br>healthy<br>strong<br>diabadass	spine<br>pain<br>chiropractic<br>backpain<br>muscles<br>smile<br>yoga<br>life<br>back<br>massage	selfie<br>migraines<br>migraine<br>ouch<br>sick<br>blue<br>anxiety<br>goodnight<br>sleep<br>blackandwhite	health<br>headache<br>headaches<br>migraines<br>stress<br>detox<br>fatigue<br>allergies<br>cramps<br>energy
**Caption**	pain<br>hope<br>feel<br>i've<br>chronic<br>don't<br>bad<br>time<br>day<br>illness	cancer<br>breast<br>awareness<br>month<br>pink<br>support<br>women<br>chemo<br>fight<br>family	i've<br>blood<br>life<br>diabetes<br>type<br>sugar<br>friends<br>make<br>high<br>live	back<br>pain<br>spine<br>neck<br>important<br>lower<br>shoulder<br>core<br>stretch<br>spinal	pain<br>migraine<br>today<br>feeling<br>migraines<br>days<br>head<br>headache<br>morning<br>work	body<br>stress<br>stomach<br>headaches<br>taking<br>works<br>headache<br>helps<br>years<br>tea
**Image**	posing<br>mammal<br>wall<br>bed<br>seat<br>hat<br>hand<br>domesticcat<br>close<br>sofa	group<br>standing<br>people<br>beautiful<br>crowd<br>little<br>girl<br>wearing<br>white<br>pink	posing<br>different<br>man<br>sitting<br>spectacles<br>sleeping<br>glass<br>line<br>alcohol<br>container	wall<br>animal<br>group<br>floor<br>sky<br>posing<br>woman<br>red<br>water<br>wooden	close<br>drink<br>hair<br>room<br>dark<br>decorated<br>indoor<br>carseat<br>open<br>sunset	woman<br>food<br>plant<br>clothing<br>white<br>spectacles<br>window<br>fruitdrink<br>half<br>snackfood

Diet (Page 1)

	Food	*Healthy food (3)*	*Healthy food (68)*	*Meat*	*Desserts*	*Vitamins & supplements*
**Hashtags**	foodporn<br>foodie<br>food<br>instafood<br>yummy<br>delicious<br>foodphotography<br>foodgasm<br>foodstagram<br>cravings	healthy<br>vegan<br>food<br>healthyfood<br>plantbased<br>vegansofig<br>vegetarian<br>foodie<br>instafood<br>eatclean	healthy<br>vegan<br>organic<br>glutenfree<br>eatclean cleaneating<br>nutrition<br>healthyfood<br>paleo<br>fruit	ribs<br>foodporn<br>food<br>bbq<br>cured<br>meat<br>foodie<br>friends<br>beef<br>pork	food<br>cravings<br>foodporn<br>delicious<br>chocolate<br>foodie<br>yummy<br>dessert<br>sweet<br>yum	vitamins<br>health<br>wellness<br>energy<br>vegan<br>detox<br>nutrition<br>supplements<br>guthealth<br>natural
**Caption**	food<br>good<br>make<br>love<br>likes<br>delicious<br>yum<br>yummy<br>amazing<br>weekend	chicken<br>dinner<br>eat<br>soup<br>rice<br>salad<br>meal<br>garlic<br>cheese<br>delicious	coconut<br>add<br>cup<br>water<br>oil<br>milk<br>honey<br>salt<br>recipe<br>ingredients	ribs<br>order<br>make<br>free<br>good<br>bbq<br>pic<br>dinner<br>great<br>nice	chocolate<br>cream<br>pumpkin<br>eat<br>good<br>made<br>butter<br>ice<br>cake<br>peanut	health<br>system<br>body<br>immune<br>vitamin<br>healthy<br>benefits<br>helps<br>natural<br>vitamins
**Image**	dish<br>meal<br>meat<br>piecederesistance<br>plate<br>sliced<br>table<br>piece<br>containing<br>arranged	meat<br>dish<br>vegetable<br>meal<br>containing<br>piecederesistance<br>cooked<br>bowl<br>sauce<br>toppings	fruit<br>dessert<br>fresh<br>sliced<br>food<br>plate<br>table<br>slice<br>containing<br>soup	dish<br>meat<br>barbecue<br>cooked<br>piecederesistance<br>pan<br>cooking<br>grill<br>sandwich<br>slice	dessert<br>chocolate<br>slice<br>food<br>piece<br>eaten<br>cream<br>plate<br>fork<br>pastry	beverage<br>vegetable<br>indoor<br>fresh<br>plant<br>arranged<br>plastic<br>fruit<br>different<br>fruitdrink

Diet (Page 2)

	Dieting & weight loss	*Weight loss (82)*	*Weight loss (94)*	*Eating disorders*	*Caffeine*	*Alcohol*
**Hashtags**	fitness<br>weightloss<br>nutrition<br>diet<br>healthy<br>fitfam<br>protein<br>dieting<br>fit<br>workout	weightloss<br>weightlossjourney<br>fitfam<br>wls<br>transformation<br>gastric<br>wlscommunity<br>weightlosstransformation<br>gastricbypass<br>bariatricsurgery	weightloss<br>diet<br>healthy<br>dieting<br>fitfam<br>weightlossjourney<br>food<br>healthyeating<br>fitness<br>exercise	recovery<br>anorexia<br>edrecovery<br>eatingdisorder<br>ana<br>eatingdisorderrecovery<br>edwarrior<br>ed<br>anorexiarecovery<br>prorecovery	caffeine<br>coffee<br>coffeelover<br>coffeeaddict<br>coffeetime<br>coffeeholic<br>hot<br>drink<br>cafe<br>coffeegram	alcohol<br>thirsty<br>drinks<br>beer<br>cocktails<br>wine<br>drink<br>cocktail<br>bar<br>yummy
**Caption**	food<br>eating<br>eat<br>weight<br>diet<br>fat<br>body<br>protein<br>healthy<br>meal	amazing<br>congrats<br>great<br>good<br>weight<br>i've<br>work<br>i'll<br>lbs<br>proud	week<br>back<br>days<br>morning<br>tonight<br>weeks<br>healthy<br>don't<br>bit<br>weekend	day<br>i've<br>today<br>hope<br>eating<br>feel<br>recovery<br>don't<br>proud<br>strong	coffee<br>day<br>tea<br>today<br>love<br>drink<br>cup<br>hot<br>green<br>feeling	drink<br>glass<br>beer<br>water<br>wine<br>make<br>cheers<br>juice<br>bottle<br>bar
**Image**	meal<br>dish<br>vegetable<br>plate<br>different<br>fresh<br>table<br>containing<br>wall<br>snackfood	dish<br>standing<br>indoor<br>meat<br>meal<br>pan<br>eaten<br>smiling<br>container<br>different	meat<br>meal<br>table<br>breakfast<br>dinner<br>piecederesistance<br>cup<br>plate<br>potato<br>egg	plate<br>meal<br>vegetable<br>breakfast<br>table<br>sliced<br>containing<br>dish<br>eaten<br>snackfood	beverage<br>food<br>coffee<br>table<br>drink<br>coffeecup<br>breakfast<br>pastry<br>dixiecup<br>doughnut	beverage<br>glass<br>drink<br>alcohol<br>beer<br>fruitdrink<br>food<br>drinking<br>empty<br>bar

Exercise (Page 1)					
	Exercise	*Running & cardio*	*Gym / bodybuilding*	*Gym / fitness*	*Gym / fitness training*
**Hashtags**	workout<br>exercise<br>motivation<br>training<br>fit<br>fitspo<br>strong<br>bodybuilding<br>gym<br>cardio	running<br>run<br>runner<br>training<br>cardio<br>fitness<br>fit<br>exercise<br>marathon<br>sore	fitfam<br>fitspo<br>bodybuilding<br>girlswholift<br>fit<br>gym<br>gymlife<br>fitnessmotivation<br>gains<br>instafit	workout<br>fitness<br>gym<br>fit<br>exercise<br>fitfam<br>fitnessmotivation<br>fitlife<br>gymlife<br>goals	workout<br>abs<br>gym<br>muscle<br>chest<br>bodybuilding<br>cardio<br>strength<br>legs<br>squats
**Caption**	love<br>amazing<br>awesome<br>great<br>body<br>girl<br>goals<br>inspiration<br>wow<br>fitness	run<br>running<br>today<br>race<br>marathon<br>walk<br>miles<br>mile<br>finish<br>half	you’re<br>text<br>join<br>page<br>message<br>number<br>great<br>clothing<br>team<br>brand	week<br>day<br>workout<br>work<br>good<br>gym<br>hard<br>i’ve<br>today<br>start	work<br>back<br>legs<br>leg<br>strong<br>workout<br>working<br>chest<br>strength<br>session
**Image**	beautiful<br>person<br>standing<br>lady<br>female<br>clothing<br>swimsuit<br>underpants<br>phone<br>floor	ground<br>person<br>way<br>grass<br>day<br>wood<br>several<br>wooded<br>forest<br>dirt	indoor<br>woman<br>meat<br>floor<br>female<br>sport<br>swimsuit<br>cellphone<br>underwear<br>mirror	posing<br>wall<br>woman<br>vegetable<br>smiling<br>spectacles<br>sport<br>plastic<br>little<br>text	indoor<br>man<br>sport<br>barbell<br>underpants<br>exercisedevice<br>swimsuit<br>male<br>sand<br>holding

Exercise (Page 2)

	Bodybuilding	*Energy & hydration*	*Yoga*	*Weight loss (82)*	*Weight loss (94)*
**Hashtags**	bodybuilding<br>shredded<br>muscle<br>gym<br>abs<br>physique<br>veins<br>gymlife<br>bodybuilder<br>gains	health<br>fitness<br>water<br>cramps<br>dehydration<br>fit<br>energy<br>dehydrated<br>fluids<br>love	yoga<br>breathing<br>yogi<br>balance<br>yogaeverydamnday<br>meditation<br>namaste<br>ear<br>breathe<br>stretch	weightloss<br>weightlossjourney<br>fitfam<br>wls<br>transformation<br>gastric<br>wlscommunity<br>weightlosstransformation<br>gastricbypass<br>bariatricsurgery	weightloss<br>diet<br>healthy<br>dieting<br>fitfam<br>weightlossjourney<br>food<br>healthyeating<br>fitness<br>exercise
**Caption**	bro<br>man<br>nice<br>work<br>likes<br>gym<br>muscle<br>hard<br>follow<br>training	water<br>body<br>blood<br>helps<br>drink<br>energy<br>reduce<br>drinking<br>increase<br>heart	yoga<br>body<br>love<br>breathing<br>mind<br>class<br>breath<br>practice<br>pose<br>breathe	amazing<br>congrats<br>great<br>good<br>weight<br>i've<br>work<br>i'll<br>lbs<br>proud	week<br>back<br>days<br>morning<br>tonight<br>weeks<br>healthy<br>don't<br>bit<br>weekend
**Image**	man<br>underpants<br>sport<br>indoor<br>barbell<br>cellphone<br>phone<br>holding<br>exercisedevice<br>swimsuit	food<br>floor<br>red<br>beverage<br>breakfast<br>laying<br>surrounded<br>purple<br>shore<br>highway	beautiful<br>indoor<br>shore<br>tree<br>water<br>beach<br>floor<br>park<br>feet<br>silhouette	dish<br>standing<br>indoor<br>meat<br>meal<br>pan<br>eaten<br>smiling<br>container<br>different	meat<br>meal<br>table<br>breakfast<br>dinner<br>piecederesistance<br>cup<br>plate<br>potato<br>egg

Healthcare & medicine					
	Healthcare	*Nursing*	*Hospitalization*	*Health science*	*Vaccination*
**Hashtags**	medicine<br>doctor<br>nurse<br>surgery<br>medical<br>medstudent<br>hospital<br>medschool<br>doctors<br>surgeon	nurse<br>hospital<br>nurselife<br>rn<br>follow<br>picoftheday<br>work<br>inspire<br>fitness<br>nurses	sick<br>hospital<br>surgery<br>strong<br>pain<br>funny<br>smile<br>pneumonia<br>blessed<br>goodvibes	medicine<br>nature<br>antibiotics<br>cancer<br>infection<br>science<br>food<br>stress<br>symptoms<br>organic	sad<br>flu<br>aesthetics<br>vaccines<br>vaccine<br>aesthetic<br>tumblr<br>hurting<br>flushot<br>organic
**Caption**	doctor<br>medical<br>surgery<br>patients<br>patient<br>hospital<br>medicine<br>doctors<br>school<br>disease	lol<br>haha<br>omg<br>true<br>lmao<br>don’t<br>hahaha<br>yeah<br>bad<br>remember	hope<br>prayers<br>love<br>sending<br>praying<br>recovery<br>strong<br>good<br>stay<br>hospital	research<br>infection<br>found<br>antibiotics<br>symptoms<br>study<br>cell<br>bacteria<br>science<br>products	flu<br>vaccines<br>vaccine<br>people<br>shot<br>children<br>don’t<br>child<br>vaccinated<br>parents
**Image**	posing<br>indoor<br>plastic<br>room<br>wearing<br>screenshot<br>suit<br>arthropod<br>invertebrate<br>shop	posing<br>indoor<br>ground<br>lady<br>glasses<br>tan<br>doll<br>pictureframe<br>newspaper<br>headcovering	posing<br>bed<br>spectacles<br>room<br>baby<br>blue<br>little<br>ground<br>boy<br>family	indoor<br>close<br>mammal<br>people<br>beer<br>vegetable<br>holding<br>document<br>sign<br>fresh	indoor<br>hand<br>black<br>beautiful<br>meal<br>domesticcat<br>screenshot<br>holding<br>lady<br>carseat

Mental health

	Mental health	*Suicide & self-harm*	*Eating disorders*
**Hashtags**	anxiety<br>depression<br>mentalhealth<br>mentalillness<br>recovery<br>anxious<br>depressed<br>bipolar<br>ptsd<br>bpd	anxiety<br>depressed<br>sad<br>suicide<br>suicidal<br>depression<br>cutting<br>sadness<br>broken<br>selfharm	recovery<br>anorexia<br>edrecovery<br>eatingdisorder<br>ana<br>eatingdisorderrecovery<br>edwarrior<br>ed<br>anorexiarecovery<br>prorecovery
**Caption**	people<br>mental<br>anxiety<br>health<br>depression<br>illness<br>life<br>feel<br>talk<br>disorder	don't<br>feel<br>talk<br>people<br>hate<br>i've<br>stop<br>anymore<br>fucking<br>cry	day<br>i've<br>today<br>hope<br>eating<br>feel<br>recovery<br>don't<br>proud<br>strong
**Image**	posing<br>indoor<br>plant<br>smiling<br>nature<br>white<br>screenshot<br>hairpiece<br>way<br>blackboard	close<br>dark<br>woman<br>staring<br>clouds<br>hand<br>cloudy<br>road<br>nightsky<br>mountain	plate<br>meal<br>vegetable<br>breakfast<br>table<br>sliced<br>containing<br>dish<br>eaten<br>snackfood

Musculoskeletal health & dermatology

	Musculoskeletal pain	*Massage*	*Injuries & rehabilitation*	*Skin health*	*Tanning*	*Cosmetic surgery*	*Dental health*
**Hashtags**	spine<br>pain<br>chiropractic<br>backpain<br>muscles<br>smile<br>yoga<br>life<br>back<br>massage	massage<br>pain<br>relax<br>arthritis<br>aches<br>therapy<br>wellness<br>painrelief<br>tension<br>relaxation	rehab<br>recovery<br>injury<br>crossfit<br>ankle<br>knee<br>physicaltherapy<br>athlete<br>strength<br>sports	skincare<br>acne<br>skin<br>beauty<br>organic<br>antiaging<br>pimples<br>clearskin<br>facial<br>eczema	sunscreen<br>love<br>sun<br>nofilter<br>summer<br>beauty<br>sunburn<br>viral<br>beach<br>organic	beauty<br>surgery<br>clinic<br>skin<br>face<br>treatment<br>aesthetics<br>doctor<br>plasticsurgery<br>surgeon	dentist<br>dental<br>smile<br>teeth<br>dentistry<br>tooth<br>toothache<br>surgery<br>odontologia<br>dentista
**Caption**	back<br>pain<br>spine<br>neck<br>important<br>lower<br>shoulder<br>core<br>stretch<br>spinal	pain<br>massage<br>body<br>muscle<br>muscles<br>relief<br>deep<br>arthritis<br>sore<br>aches	knee<br>ankle<br>injury<br>foot<br>pain<br>recovery<br>leg<br>training<br>hip<br>surgery	skin<br>acne<br>products<br>face<br>cream<br>oil<br>natural<br>mask<br>product<br>dry	hair<br>sun<br>sunscreen<br>light<br>free<br>pretty<br>face<br>side<br>city<br>hand	call<br>results<br>treatment<br>surgery<br>info<br>nose<br>clinic<br>patient<br>consultation<br>email	teeth<br>smile<br>tooth<br>dental<br>mouth<br>dentist<br>treatment<br>patient<br>case<br>office
**Image**	wall<br>animal<br>group<br>floor<br>sky<br>posing<br>woman<br>red<br>water<br>wooden	indoor<br>close<br>man<br>floor<br>decorated<br>wearing<br>half<br>hotel<br>honey<br>cake	floor<br>people<br>outdoor<br>feet<br>sport<br>ceiling<br>wall<br>field<br>shoes<br>indoor	posing<br>several<br>beverage<br>arranged<br>lotion<br>smiling<br>hair<br>green<br>staring<br>vectorgraphics	posing<br>shore<br>sky<br>beach<br>sitting<br>hairpiece<br>headdress<br>sand<br>set<br>swimming	posing<br>hair<br>woman<br>female<br>spectacles<br>room<br>clothing<br>young<br>clouds<br>cosmetic	posing<br>close<br>dessert<br>plastic<br>toothbrush<br>eyes<br>cosmetic<br>floor<br>brush<br>mouth

Sleep		
	Sleep	*Insomnia*
**Hashtags**	bedtime<br>instagood<br>tired<br>photooftheday<br>sleep<br>dark<br>sleepy<br>bed<br>goodnight<br>night	insomnia<br>bedtime<br>workout<br>art<br>selfie<br>rest<br>night<br>natural<br>sleepy<br>amazing
**Caption**	love<br>cute<br>haha<br>bed<br>time<br>omg<br>miss<br>nice<br>cool<br>adorable	sleep<br>night<br>bed<br>hours<br>time<br>back<br>asleep<br>sleeping<br>make<br>nights
**Image**	laying<br>mammal<br>bed<br>girl<br>bedclothes<br>young<br>pillow<br>night<br>red<br>brown	indoor<br>lying<br>bedclothes<br>smiling<br>pillow<br>blanket<br>sofa<br>bedroom<br>glasses<br>cloth

Substance use

	Marijuana	*Caffeine*	*Alcohol*
**Hashtags**	cannabis<br>chronic<br>marijuana<br>weed<br>weedstagram<br>cannabiscommunity<br>thc<br>dank<br>hightimes<br>dabs	caffeine<br>coffee<br>coffeelover<br>coffeeaddict<br>coffeetime<br>coffeeholic<br>hot<br>drink<br>cafe<br>coffeegram	alcohol<br>thirsty<br>drinks<br>beer<br>cocktails<br>wine<br>drink<br>cocktail<br>bar<br>yummy
**Caption**	check<br>cannabis<br>high<br>cbd<br>smoke<br>stay<br>fire<br>marijuana<br>weed<br>product	coffee<br>day<br>tea<br>today<br>love<br>drink<br>cup<br>hot<br>green<br>feeling	drink<br>glass<br>beer<br>water<br>wine<br>make<br>cheers<br>juice<br>bottle<br>bar
**Image**	vegetable<br>close<br>plant<br>hand<br>eaten<br>piece<br>leaf<br>half<br>tree<br>palm	beverage<br>food<br>coffee<br>table<br>drink<br>coffeecup<br>breakfast<br>pastry<br>dixiecup<br>doughnut	beverage<br>glass<br>drink<br>alcohol<br>beer<br>fruitdrink<br>food<br>drinking<br>empty<br>bar
